# Increasing H2B Monoubiquitination Improves the Transcriptome and Memory in the Aged Hippocampus

**DOI:** 10.1523/ENEURO.0037-25.2025

**Published:** 2025-04-11

**Authors:** Shannon Kincaid, Gueladouan Setenet, Natalie J. Preveza, Kaiser C. Arndt, Phillip Gwin, Yu Lin, Hehuang Xie, Timothy J. Jarome

**Affiliations:** ^1^School of Animal Sciences, Virginia Polytechnic Institute and State University, Blacksburg, Virginia 24061; ^2^School of Neuroscience, Virginia Polytechnic Institute and State University, Blacksburg, Virginia 24061; ^3^Department of Biomedical Sciences and Pathobiology, Virginia Polytechnic Institute and State University, Blacksburg, Virginia 24061; ^4^Fralin Life Science Institute, Virginia Polytechnic Institute and State University, Blacksburg, Virginia 24061

**Keywords:** aging, hippocampus, histone H2B, monoubiquitination, transcriptome

## Abstract

A decline in cognitive abilities is associated with the aging process, affecting nearly 33% of US adults over the age of 70, and is a risk factor for the development of dementia and Alzheimer's disease. Several studies have reported age-related alterations in the transcriptome in the hippocampus, a major site of memory storage that is among the first regions impacted with age, dementia, and Alzheimer's disease. However, much remains unknown about why these transcriptional changes exist in the aged hippocampus and how this impacts memory late in life. Here, we show that monoubiquitination of histone H2B (H2Bubi), an epigenetic mechanism recently reported to be major regulator of the epigenome and transcriptome during memory formation in the young adult brain, decreases with age in the hippocampus of male rats. In vivo CRISPR-dCas9–mediated upregulation of *Rnf20*, the only ubiquitin E3 ligase for H2B, in the hippocampus significantly improved memory retention in aged rats. Remarkably, RNA-seq analysis revealed that in addition to the 18 genes typically upregulated in the aged rat hippocampus following contextual fear conditioning, *Rnf20* upregulation caused learning-related increases and decreases in 40 and 11 unique genes, respectively, suggesting that these 51 genes may be among those most critical for improving memory in advanced age. Together, these data suggest that H2B monoubiquitination is a significant regulator of age-related dysregulation of the transcriptome and impairments in memory.

## Significance Statement

Age-related memory decline impacts the lives of millions of Americans and is a risk factor for developing dementia. It is imperative that we understand why brain molecular mechanisms change with age in order to reverse memory loss late in life. Here we show that changes in levels of a major epigenetic modification, histone H2B monoubiquitination (H2Bubi), in the hippocampus impacts memory late in life. Importantly, memory and gene expression can be improved in the aged hippocampus through the upregulation of H2Bubi ligase, *Rnf20*, using CRISPR-dCas9. This research gives insight into how gene expression in the hippocampus changes with age and leads to memory decline.

## Introduction

Age-related memory loss affects millions of Americans every year, and ∼6.9 million people over the age of 65 are diagnosed with Alzheimer's disease (Humphreys, 2012). The hippocampus, which is among the first brain regions impacted in dementia and Alzheimer's disease, is important for transforming labile short-term memories (STMs) to stable long-term memories (LTMs; Apostolova et al., 2012; Squire et al., 2015; Ramos Bernardes da Silva Filho et al., 2017). Consequently, numerous studies have suggested that dysregulation of a variety of molecular processes within the hippocampus may be one of the primary contributors to age-related memory decline (Rosenzweig and Barnes, 2003; Kawahara et al., 2009; Hullinger and Puglielli, 2017; Patrick et al., 2024). Of these, age-related changes in gene transcription have received significant attention, which has often been correlated with dysregulation of epigenetic mechanisms that have known roles in memory formation in the young adult brain (Jiang et al., 2001; Liu et al., 2009; Ianov et al., 2017; Creighton et al., 2020; Maity et al., 2021). However, to date, much remains unknown about why these changes in the epigenome and transcriptome occur in the hippocampus late in life.

Epigenetic mechanisms can be broadly defined into DNA methylation and histone post-translational modifications (Kim and Kaang, 2017; Jarome et al., 2021), the latter of which can activate or repress gene transcription depending on the type of modification that occurs (Fischer et al., 2007; López et al., 2020; Jarome et al., 2021; Maity et al., 2021). While there are a variety of histone modifications, the most studied are typically chemical modifications such as acetylation and methylation (Bannister and Kouzarides, 2011), both of which have been shown to be altered in the aged hippocampus and correlate with or contribute to age-related memory loss (Peleg et al., 2010; Morse et al., 2015; Nativio et al., 2018). However, histone tails can also be modified by other proteins, of which the most common is attachment of a single ubiquitin protein via ubiquitination. As the proteasome has low affinity for monoubiquitination, this modification of histones does not lead to protein degradation but instead is involved in regulating the chromatin structure and DNA accessibility. Consistent with this, monoubiquitination of H2B at lysine-120 (H2Bubi) is associated with transcriptional activation and repression while monoubiquitination of H2A at lysine-119 (H2Aubi) is typically only associated with gene silencing (Tanny et al., 2007; Zhou et al., 2008; Fuchs et al., 2012; Srivastava et al., 2017).

While rarely studied in the brain, to date, recent evidence suggests an important role for histone ubiquitination in the form of H2Bubi in memory formation in the hippocampus. H2Bubi increases in the young adult hippocampus following contextual fear conditioning and loss of *Rnf20*, the only ubiquitin ligase for H2B, abolishes learning-induced increases in active histone methylation and impairs synaptic plasticity and memory formation (Jarome et al., 2021). Interestingly, one recent study found that loss of H2Bubi in the hippocampus completely abolished the learning-related transcriptome, including all gene upregulation and downregulation that occurred following contextual fear conditioning (Navabpour et al., 2024). Together, these studies indicate that H2Bubi may be a critical regulator of the transcriptome necessary for normal memory formation in the hippocampus. This raises the interesting possibility that changes in H2Bubi levels may contribute to transcriptional dysregulation and memory impairments associated with advanced age. However, to date, H2Bubi has never been studied within the context of the aging brain.

In this study, we investigated the role of H2Bubi in memory during advanced age. We found that H2Bubi levels decrease in the aged hippocampus. Reversing this via CRISPR-dCas9–mediated upregulation of *Rnf20* in the hippocampus improved memory in aged rats and resulted in a more robust transcriptome following learning. Together, these data indicate that reductions in H2Bubi may be a major contributor to transcriptional dysregulation and memory impairments late in life.

## Materials and Methods

### Subjects

Experiments used 3, 12, and 22–24 month-old (m.o.) male Fischer rats obtained from the National Institute on Aging (NIA) animal colony at Charles River Laboratories. Aged female rats were not used as they were not available from the vendor during the time these experiments were completed. Animals were housed two per cage with *ad libitum* access to water and rat chow, the colony maintained under a 12 h light/dark cycle, and all experiments took place during the light portion of the cycle. All procedures were approved by the Institutional Animal Care and Use Committee and conducted with the ethical guidelines of the National Institutes of Health.

### CRISPR-dCas9 intracranial injections

We used a CRISPR guide RNA (gRNA) targeting a 200 bp region within the *Rnf20* promoter that was previously designed, cloned, and validated in vivo (Jarome et al., 2021). For stereotaxic delivery, Rnf20-gRNA alone (Control) or with dCas9-VPR (#63798, Addgene) plasmids were mixed with in vivo-jetPEI (Polyplus Transfection) according to the manufacturer's instructions. Rats underwent stereotaxic surgeries under anesthesia with 2–4% isoflurane where these plasmids were bilaterally injected into the CA1 region of the dorsal hippocampus (CA1) using coordinates relative to bregma (A/P: −3.6, M/L: ±1.7, D/V: −3.6) at a rate of 0.1 μl per minute for a total of 1.0 μl per hemisphere. Animals received a subcutaneous injection of carprofen (5 mg/kg) and topical lidocaine on the day of surgery. Subcutaneous carprofen (5 mg/kg) injections were administered once per day for 2 d following surgery.

### Apparatus

This project used two identical fear conditioning chambers that consisted of a steel test cage with front and back Plexiglas walls and a grid shock floor above a plastic drop pan. The top of the chamber had a house light, which remained on during the behavioral procedures. A USB camera was mounted on a steel panel outside the back Plexiglas wall of the chamber, angled at ∼45°. The entire chamber was housed in an isolation cubicle with an acoustic liner and a house fan, which remained active during all behavioral procedures. Shock was delivered through the grid floor via a Precision Animal Shocker under the control of FreezeFrame 4 software, which also analyzed animal behavior in real time. A freezing threshold of 2.0 was used as the scoring parameter for all animals. All videos were recorded and stored for later analysis. The chamber walls were wiped with 70% isopropanol before use.

### Behavioral procedures

Rats underwent a contextual fear conditioning procedure. Animals were first handled for 4 d prior to behavioral training. On the training day, animals were placed into the fear conditioning apparatus and after a 1 min baseline, received 4 unsignaled footshock (1.0 mA, 1 s) presentations, followed by a 1 min postshock period, after which animals were returned to their home cage. Memory retention testing was conducted 24 h later, during which animals were placed back into the chamber for 5 min in the absence of footshock presentations before being returned to their home cage.

### Tissue collection

Rats were overdosed on isoflurane in a necrosis chamber, decapitated, and then the brain was rapidly removed and immediately frozen on dry ice. Both hemispheres of the dorsal CA1 region were then dissected out blocking the brain in a rat brain matrix (Harvard Apparatus) incubated with dry ice. All dissected tissue was frozen at −80°C until needed.

### Histone extractions

Tissues were homogenized in nondenaturing sucrose buffer and subjected to centrifugation at 7,700 × *g* for 1 min. The pellet containing nuclei was resuspended in 250 µl of 0.4 N sulfuric acid and then incubated on ice for 30 min followed by centrifugation at 14,000 × *g* for 30 min at 4°C. The resulting supernatant was mixed with trichloroacetic acid with 4 mg/ml deoxycholic acid and incubated on ice for 30 min. Precipitated protein was recovered by centrifugation and followed by acetone drying. All procedures were carried out under ice-cold conditions. The purified histone enriched protein pellet was resuspended in 10 mM Tris, pH 8.0. Protein concentrations were determined by the Bradford assay (Bio-Rad).

### Antibodies

Antibodies included monoubiquitinated histone H2B (1:1,000, #5546, Cell Signaling Technology) and total histone H3 (1:1,000, #1791, Abcam).

### Western blot

Normalized proteins (3 µg) were separated on a 20% polyacrylamide gel with a 4% stacking gel before being transferred onto an Immobilon-FL membrane using the turbo transfer system (Bio-Rad). Membranes were washed in TBS plus 0.1% Tween-20 (TBSt) and then blocked in a 50:50 LI-COR blocking buffer (50% LI-COR TBS blocking buffer and 50% TBSt) for 1 h at room temperature. After blocking, membranes were incubated overnight in primary antibody at 4°C. The following day, membranes were washed three times with TBSt for 10 min, incubated in a Goat anti-Rabbit secondary antibody (1:40,000; LI-COR) for 45 min, and then washed twice in TBSt for 10 min. Membranes were rinsed with TBS before imaging with the Odyssey Fc near infrared system (LI-COR). After each development, membranes were stripped for 10 min with 0.2 N NaOH, washed with TBSt for 15 min twice, and then reblocked for 1 h before overnight incubation in primary antibody at 4°C. Image Studio ver 5.2. was used to quantify proteins. Mean pixel density was calculated for each sample and normalized to histone H3, which was used as a loading control, and expressed as a percentage of the control group.

### RNA extractions

RNA was extracted with the RNeasy Mini Kit (Qiagen) following the manufacturer instructions. RNA concentration and quality were determined by NanoDrop.

### RNA sequencing

Total RNA (150 ng) was extracted from each tissue sample and sent to Novogene Corporation Inc. (Sacramento, CA, USA) for RNA sequencing (RNA-seq) and library preparation. Libraries were constructed and sequenced on the Illumina NovaSeq X Plus platform using 150 bp paired-end reads (Illumina, San Diego, CA, USA). Quality control steps involved removing raw reads that met the following criteria: presence of adapter sequences, reads with more than 10% unidentified nucleotides (*N* > 10%), and low-quality reads where over 50% of the bases had a Q score ≤ 5. High-quality clean reads were aligned to the GRCr8 reference genome using HISAT2 (Sirén et al., 2014).

### Differentially expressed genes analysis and gene ontology

Gene expression levels were quantified, and differential gene expression analysis was performed using the DESeq2 (Love et al., 2014) package in R. Differentially expressed genes (DEGs) were identified based on a fold change greater than 1.2 and an adjusted *p*-value less than 0.05. Gene ontology (GO) enrichment analysis for biological processes was conducted using the clusterProfiler (Yu, 2024) package (v4.12.6) in R with default parameters. Visualization of enriched GO terms was also carried out using clusterProfiler.

### Chromatin immunoprecipitation

For chromatin immunoprecipitation (ChIP), one hemisphere of CA1 tissue was ﬁxed in PBS with 1% formaldehyde for 10 min at 37°C and then 2.5 M glycine was added to quench the reaction. Fixed tissue was then extensively washed in PBS before being homogenized in hypotonic buffer (10 mM KCl, 20 mM HEPES, 1 mM MgCl, 1 mM DTT) with protease inhibitors. Homogenates were centrifuged at 1,350 g for 10 min at 4°C and the resulting pellets (nuclei) were resuspended in ChIP sonication buffer (1× TE with 1% SDS) with protease inhibitors. Chromatin was sheared to ∼300 bp using the QSonic 800R2 Sonicator with 55% amplitude and 20 s pulse for 35 min. Following sonication, samples were centrifuged at 20,000 g for 10 min at 4°C and then the supernatant (DNA) was collected and DNA concentration was measured using a NanoDrop. DNA amount was normalized by dilution with TE buffer and 2× RIPA buffer (2× PBS, 1% sodium deoxycholate, 2% NP-40, 0.2% SDS) with 2× proteasome inhibitors. Normalized DNA samples were then combined with Magna ChIP protein A/G magnetic beads (#16-663, MilliporeSigma). Immunoprecipitations were carried out overnight at 4°C with primary antibody (anti-H2BubiK120, 5 μg) or no antibody (control). The next day, immune complexes were washed with low-salt buffer (20 mM Tris at pH 8.0, 0.1% SDS, 1% Triton X-100, 2 mM EDTA, 150 mM NaCl), high-salt buffer (20 mMTris at pH 8.1, 0.1% SDS, 1% Triton X-100, 500 mM NaCl, 1 mM EDTA), and LiCl immune complex buffer (0.25 M LiCl, 10 mM Tris at pH 8.1, 1% deoxycholic acid, 1% IGEPAL-CA630, 500 mM NaCl, 2 mM EDTA), and twice with TE buffer, and then extracted in TE containing 1% SDS and sodium bicarbonate. The samples were then heated overnight at 65°C to reverse protein–DNA cross-links, and then proteins were destroyed with proteinase K digestion (100 µg for 2 h at 37°C). Phenol/chloroform/isoamyl alcohol and then ethanol precipitation were used to isolate DNA, which was used for quantitative real-time PCR with the following primers speciﬁc to the rat *LOC120093683* promoter Forward 3: GCTGTCGGAAAGCTCTGGTA Reverse 3: AGCTAGGCCATTAATGTGGGA. Quantitative PCR ampliﬁcations of ChIP DNA were performed on the Bio-Rad CFX96 real-time system using the following protocol: 3 min at 95.0°C, then 10 s at 95.0°C, followed by 30 s at 60°C (39 repeats), followed by a melt curve starting at 55.0°C for 10 s (81 repeats). For analysis, the cumulative ﬂuorescence for each amplicon was taken as a percentage of the enrichment and taken as a fold change of the control group.

### Statistical analyses

Data are presented as mean and standard error with scatterplots of individual values (except in line graphs). Data were analyzed using independent-samples *t* tests or one-way or two-way ANOVA and Tukey's multiple-comparisons post hoc tests using Prism software (GraphPad Software). All experimenters were blinded to treatment groups. The Brown–Forsythe test was used to determine normal distribution of data.

## Results

### Aging results in loss of H2Bubi in the hippocampus

**Figure 1.**
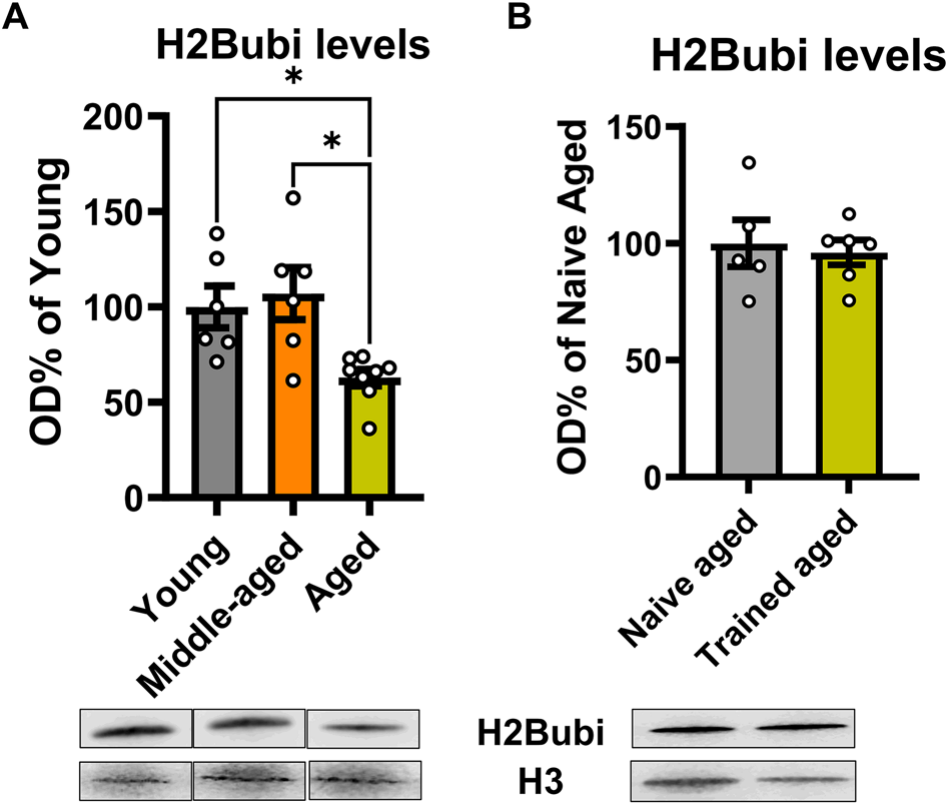
H2Bubi is reduced in the aged hippocampus. ***A***, We compared baseline levels of H2Bubi in the dorsal CA1 region of the hippocampus in young adult (3 m.o), middle-aged (12 m.o), and aged (24 m.o.) male rats (*N* = 6–8 per group). H2Bubi levels were significantly reduced in the hippocampus of aged rats compared with young adult and middle-aged. ***B***, Aged (24 m.o.) male rats were trained to contextual fear conditioning and killed 1 h later. H2Bubi levels in fear-conditioned animals were similar to naive controls (*N* = 6 per group). *p < 0.05.

We first tested if H2Bubi levels change with age in the hippocampus. To do this, we compared the expression of H2Bubi in the dorsal CA1 region of young adult (3 m.o), middle-aged (12 m.o.), and aged (24 m.o.) male rats (Fig. 1*A*). We found a main effect for age (*F*_(2,17)_ = 6.776, *p* = 0.0069). Post hoc tests revealed that H2Bubi levels decreased in the aged CA1 region relative to both young adult (*p* = 0.0303) and middle-aged animals (*p* = 0.0101). Considering this reduction and that prior studies report ∼25% increase in H2Bubi levels in the hippocampus of young adult animals following behavioral training (Jarome et al., 2021), we next tested whether aged rats would show an increase in H2Bubi in the hippocampus after contextual fear conditioning (Fig. 1*B*). For this, we compared aged naive animals with aged fear-conditioned rats in which CA1 tissue was collected 1 h later (Jarome et al., 2021). Notably, there was no change in H2Bubi levels (*t*_(9)_ = 0.3645, *p* = 07239) between naive aged rats and the contextual fear trained aged rats. Together, these data suggest that aging results in reductions in H2Bubi levels in the hippocampus as well as an altered response of this epigenetic mark following learning.

### Upregulation of the H2Bubi ligase *Rnf20* in the hippocampus improves memory in aged rats

**Figure 2.**
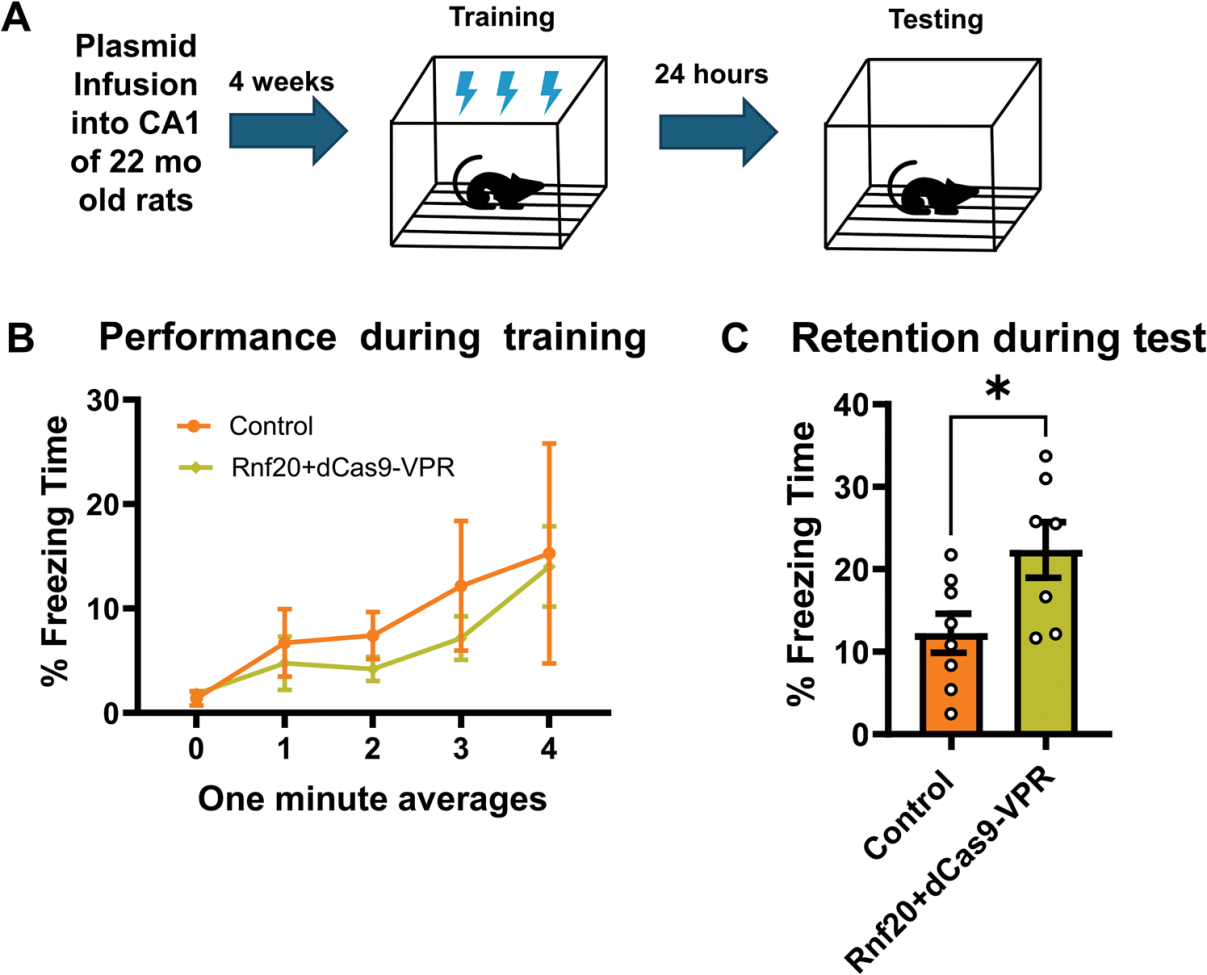
Upregulation of the H2Bubi ligase *Rnf20* in the aged hippocampus improves contextual fear memory. ***A***, Aged (22 m.o) male rats (*N* = 7–8 per group) were stereotaxically injected with a CRISPR-dCcas9-VPR plus *Rnf20* gRNA to upregulate expression of the E3 ligase RNF20 responsible for monoubiquitinating histone H2B. ***B***, During training we saw no significant differences in performance. ***C***, However, rats that received the CRISPR-dCas9–mediated upregulation of *Rnf20* showed markedly better memory retention during testing than the control group. *p < 0.05.

We next wanted to test if increasing H2Bubi in the aged hippocampus can improve memory late in life. Previous work has shown that CRISPR-dCas9–mediated increases in the H2Bubi ligase *Rnf20* can promote H2Bubi and enhance memory in the young adult hippocampus (Jarome et al., 2021). Therefore, we infused a dCas9-VPR transcriptional activator plasmid in conjunction with the prior developed *Rnf20* gRNA into the dorsal CA1 region of 22 m.o. male rats. Four weeks later, animals were trained and tested to contextual fear conditioning (Fig. 2*A*). During training we found a main effect for time (*F*_(4,52)_ = 4.015, *p* = 0.0065), but not manipulation (*F*_(1,13)_ = 0.3068, *p* = 0.5891) and time × manipulation interaction (*F*_(4,52)_ = 0.1758, *p* = 0.9499; Fig. 2*B*) However, during test we found that upregulation of *Rnf20* significantly improved memory retention relative to control (*t*_(13)_ = 2.499, *p* = 0.0266; Fig. 2*C*). This indicates that promoting H2Bubi significantly improves memory in aged rats.

### *Rnf20* upregulation increases the training-induced transcriptome in the dorsal hippocampus of aged rats

It has been previously shown that H2Bubi is a major regulator of the transcriptome during memory formation (Navabpour et al., 2024). Therefore, we next tested if upregulation of H2Bubi impacts the transcriptome in aged rats during memory formation. We infused aged (22 m.o.) male rats with *Rnf20*-gRNA + dCas9-VPR or control. Animals were trained to contextual fear conditioning and killed 1 h later, after which the CA1 region of the dorsal hippocampus was collected and compared with control-infused naive animals using whole genome RNA sequencing.

**Figure 3.**
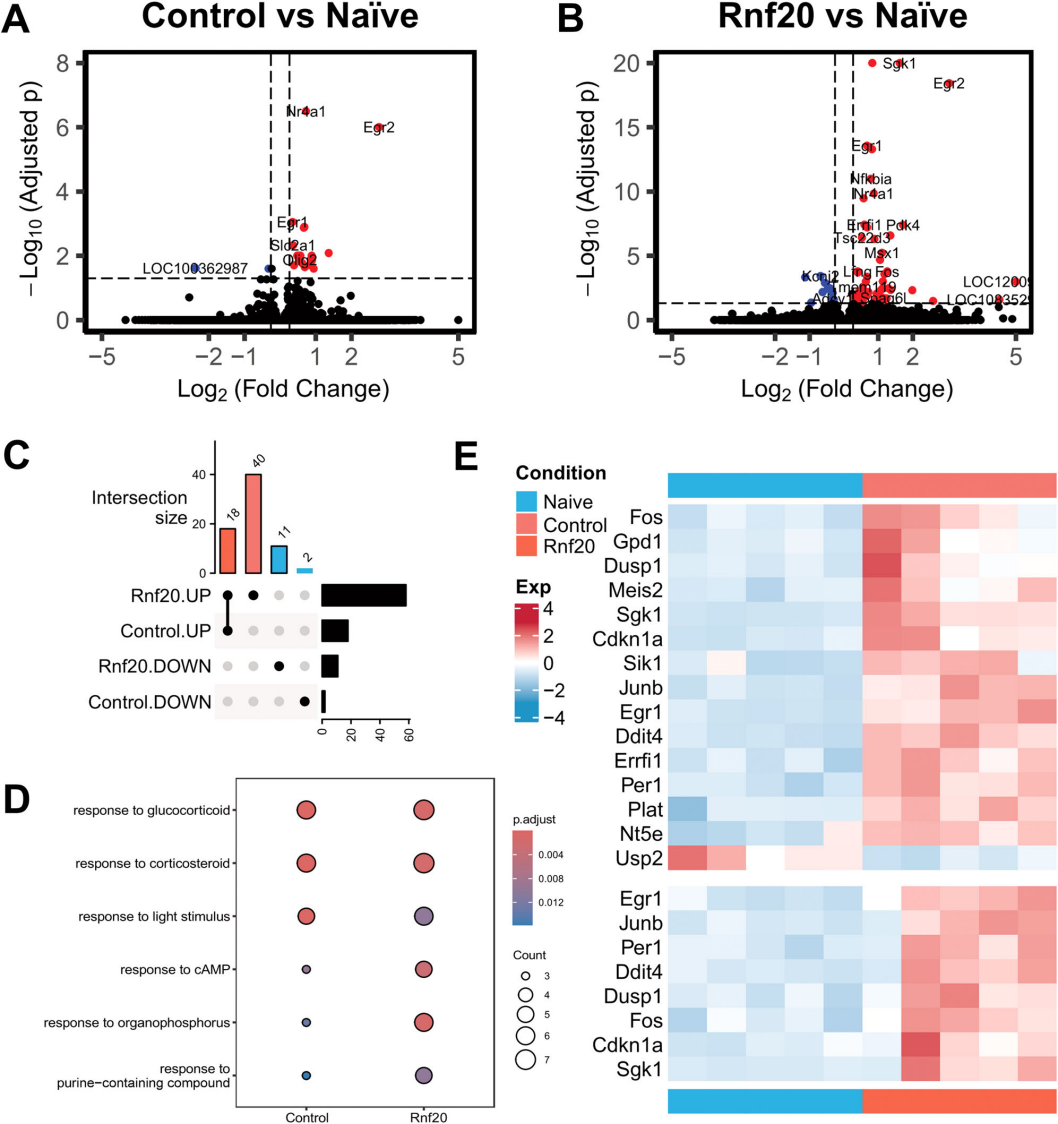
Upregulation of H2Bubi ligase *Rnf20* in aged hippocampus improves the learning-related transcriptome. Using whole genome RNA sequencing, we compared differential gene expression in the CA1 region of the dorsal hippocampus between aged fear-conditioned-control and fear-conditioned *Rnf20*-gRNA + dCas9-VPR groups relative to control-injected naive animals (*N* = 5 per group). CA1 tissue was collected 1 h after training. ***A***, Volcano plot of DEGs for the trained-control group relative to naive-control animals. ***B***, Volcano plot of DEGs of the trained *Rnf20*-gRNA + dCas9-VPR group relative to naive-control animals. ***C***, There were 18 common upregulated DEGs between the control-trained and trained-*Rnf20*-siRNA groups. However, there were 51 DEGs unique to the trained *Rnf20*-gRNA + dCas9-VPR, 40 being upregulated and 11 downregulated. ***D***, Pathway analysis identifying differences between trained *Rnf20*-gRNA + dCas9-VPR and trained-control groups showing the increases in genes in different pathways. ***E***, Heat map displaying the expression levels of DEGs across conditions. Please see Extended Data Figure 3-1 for extended data.

Next, we performed differential gene expression (DEG) analysis to compare transcriptional differences across conditions. All significantly altered genes are presented in Table 1. First, we specifically analyzed the trained-control (Control) and trained-*Rnf20*-treatment (Rnf20) groups versus the naive animals. For the trained-control condition, we identified 20 DEGs, including 18 upregulated genes and 2 downregulated genes (Fig. 3*A*). While this is a low number of genes compared with prior studies examining the rat hippocampus under similar conditions and approaches (Farrell et al., 2024), this would be consistent with the poor memory typically seen in aged rats. In the trained-*Rnf20*-treatment group we identified 69 DEGs, comprising 58 upregulated and 11 downregulated genes (Fig. 3*B*). Next, we investigated the overlap between the DEGs identified in the two conditions. Remarkably, the 18 upregulated genes in the trained-control group were shared in the *Rnf20* manipulation, though 40 new genes were increased in the *Rnf20* upregulation group (Fig. 3*C*). This overlap suggests that the upregulation of *Rnf20* induces not only the same transcriptional changes to those observed with training alone but results in a more robust transcriptome that includes 51 new genes (40 upregulated and 11 downregulated). When comparing trained-control against trained-*Rnf20*-treament groups, we did not observe any significant DEGs, though this would be expected as learning did drive at least partially overlapping gene expression profiles in both conditions (Fig. 3*C*). Using ChIP focused on the *LOC120093683* gene, which showed the highest fold change in the *Rnf20* group but was not significantly altered in controls, we found a main effect for manipulation (*F*_(2,12)_ = 5.303, *p* = 0.0224; Extended Data Fig. 3-1). Post hoc analysis confirmed that *Rnf20* upregulation significantly increased levels of H2Bubi relative to naive (*p* = 0.0294) animals with a strong trend for an increase compared with trained-control animals (*p* = 0.0519). These data support the restorative impact of *Rnf20* upregulation on H2Bubi targeting to specific genomic loci following learning.

10.1523/ENEURO.0037-25.2025.f3-1 Figure 3-1 **Upregulation of *Rnf20* increases H2B monoubiquitination at *LOC120093683* in the aged hippocampus.** Using chromatin immunoprecipitation analysis, we compared H2B monoubiquitination (H2Bubi) levels at *LOC120093683* in the CA1 region of the dorsal hippocampus between aged fear conditioned-control and fear conditioned *Rnf20*-gRNA+dCas9-VPR groups relative to control-injected naïve animals (N = 5 per group). CA1 tissue was collected 1 hour after training. Upregulation of *Rnf20* increased H2Bubi after training. *P < 0.05 from Naïve. #P = 0.0519 from Control Trained. Download Figure 3-1, TIF file.

**Table 1.** Differentially expressed genes in the aged hippocampus following fear conditioning

*Rnf20* trained	Both	Control trained
***Upregulated Genes***		
Acer2	Ddit4	-
Apold1	Dusp1	-
Arc	Egr1	-
Arrdc2	Egr2	-
Arrdc3	Fos	-
Cfap46	Junb	-
Clec18a	Lfng	-
Col8a1	Nfkbia	-
Dnah1	Nr4a1	-
Dnah11	Olig2	-
Dnah6	Per1	-
Dusp5	Plekhf1	-
Egr4	Prokr2	-
Elovl7	Sgk1	-
Errfi1	Slc2a1	-
Fam43a	Tmem119	-
Fgf1	Tob2	-
Foxj1	Cdkn1a	-
Fstl1	-	-
Lama3	-	-
LOC108352943	-	-
LOC120093683	-	-
Map2k3	-	-
Meis2	-	-
Msx1	-	-
Nfil3	-	-
Nt5e	-	-
Pdk4	-	-
Plat	-	-
Rgs22	-	-
Rsrp1	-	-
Sik1	-	-
Spag6l	-	-
Spred1	-	-
Sulf1	-	-
Tinagl1	-	-
Tmem212	-	-
Tsc22d3	-	-
Ttc21a	-	-
Zmynd10	-	-
Gpd1		
***Downregulated Genes***		
Ddit4l	-	LOC100362987
LOC120097130	-	*Tbrg4*
LOC103690059	-	Ryr2
Mex3b	-	-
LOC102551114	-	-
LOC102547703	-	-
Spry2	-	-
Adcy1	-	-
Erf	-	-
Usp2	-	-
Kcnj2	-	-

To further explore the biological functions of the identified DEGs, we conducted Gene Ontology (GO) enrichment analysis using the clusterProfiler package. This analysis revealed significant enrichment of several biological processes (Fig. 3*D*), including responses to glucocorticoids, cAMP, and light stimuli, among others. Notably, the upregulated genes in the *Rnf20* and controlled-trained conditions were strongly associated with stress–response pathways and signaling processes, highlighting the potential functional implications of these transcriptional changes to memory formation in advanced age. To further investigate the expression patterns of key DEGs within these pathways, we generated a heat map (Fig. 3*E*). This visualization underscores the distinct and consistent upregulation of genes such as *Egr1*, *Junb*, *Per1*, *Sgk1*, and *Fos*, all genes vital to hippocampal memory pathways (Strekalova et al., 2003; Lang et al., 2010; Duclot and Kabbaj, 2017), in both the *Rnf20* and control groups compared with the naive animals. Collectively, these findings suggest that upregulation of H2Bubi increases the learning-induced transcriptome of aged animals, which may drive the improvement in memory we observed in our prior experiment.

## Discussion

Histone post-translational modifications have been widely implicated in memory formation in the brain (Gräff and Tsai, 2013). Of these, H2Bubi has recently been shown to play a significant role in the transcriptional activation and repression of gene expression during memory formation in the young adult hippocampus (Farrell et al., 2024; Navabpour et al., 2024). However, little is known about how H2Bubi changes in the hippocampus with age and impacts the transcriptome and memory late in life. Here, we show that H2Bubi is decreased in the aged hippocampus and that upregulation of the H2Bubi ligase, *Rnf20*, improves memory and the learning-related transcriptome late in life. These data suggest that reductions in H2Bubi within the hippocampus across the lifespan may significantly contribute to age-related memory decline.

Our data indicates that H2Bubi decreases later in life, impacting gene expression in the hippocampus that may contribute to age-related memory deficits. This is consistent with previous studies indicating that H2Bubi is important in memory formation in young adult rats as it is a major regulator of transcriptional changes following learning (Navabpour et al., 2024). We found that increasing H2Bubi in the aged hippocampus not only improved memory performance but significantly changed the learning-related transcriptome. Notably, while there were 18 genes common to all fear-conditioned groups, increasing the H2Bubi ligase *Rnf20* resulted in the increased or decreased expression of an additional 51 unique genes. This is consistent with prior work that H2Bubi can regulate both transcriptional activation and repression during memory formation (Jarome et al., 2021; Navabpour et al., 2024). Many of the common genes across conditions included *Junb*, *Scl2a1*, *Fos*, and *Egr1*, all of which have been shown to be important for memory formation (Radulovic et al., 1998; Strekalova et al., 2003; Maddox et al., 2011). Conversely, the genes uniquely altered following *Rnf20* upregulation included *Dnah1*, *Pdk4*, *Clec18c*, and *Col8a1*, which are involved in structural, metabolic, and cell adherence. While these latter genes have not been well studied in the context of memory formation, this suggests that they could be critically involved in improving memory late in life. Future studies will aim to better understand the unique contribution of these genes to memory formation both early and late in life.

Our data indicates that H2Bubi levels decrease with age and that reversing this can improve the transcriptome and memory late in life. However, the reason for which H2Bubi begins to decrease with age is unknown. While not directly tested in our study, it is possible that RNF20 levels change with age, which could lead to a loss of H2Bubi. However, recent evidence indicates widespread dysregulation of the protein degradation function of the ubiquitin-proteasome system (UPS) with age (Patrick et al., 2024). This could in turn lead to secondary effects on other forms of ubiquitination due to changes in the free ubiquitin pool and alterations in ubiquitin recycling. Examining the precise relationship between age-related changes in the UPS and H2Bubi will be of interest in future studies. Further, in the young adult hippocampus H2Bubi has been shown to regulate memory formation via recruitment of histone methylation (Jarome et al., 2021). However, whether increasing H2Bubi improves memory through the recruitment of histone methylation in advanced age has yet to be determined and will be of critical importance to examine in future studies.

Although the hippocampus is highly affected by the aging process, other brain regions, such as the amygdala, prefrontal cortex, and retrosplenial cortex, are also impacted and can contribute to age-related memory decline. It is interesting to speculate that dysregulation of H2Bubi could also occur in these other brain regions across the lifespan and contribute to age-associated transcriptional dysregulation and memory decline. However, unlike other histone modifications that have been indicated to impact memory in these regions (Gräff et al., 2012; Gupta-Agarwal et al., 2014) and shown to be dysregulated in Alzheimer's disease patients (Basavarajappa and Subbanna, 2021), H2Bubi has yet to be examined outside of the hippocampus at any age. Directly testing how H2Bubi changes and interacts with other histone modifications within these brain regions during memory will give further insight into its specific role in age-related memory decline. In addition, testing its regulation of the transcriptome to determine a common mechanism across the brain for gene transcriptional control could be fundamental in developing treatment opportunities for memory deficits in late life.

In conclusion, we found that H2Bubi decreases in the hippocampus with age and that reversing this improves the transcriptome and memory late in life. These findings help further our understanding of the molecular mechanisms contributing age-related memory decline and dysregulation of the transcriptome.

## Synthesis

Reviewing Editor: Harald Sontheimer, University of Virginia Decisions are customarily a result of the Reviewing Editor and the peer reviewers coming together and discussing their recommendations until a consensus is reached. When revisions are invited, a fact-based synthesis statement explaining their decision and outlining what is needed to prepare a revision will be listed below. The following reviewer(s) agreed to reveal their identity: NONE.

Please look at the suggestions for editorial changes provided by the two referees below:

The statement "Age-related memory loss affects millions of Americans every year, and approximately 6.9 million people over the age of 65 are diagnosed with Alzheimer's disease (Humphreys, 2012)." Please provide a recent reference. This will benefit the readers.

The hippocampus, which is among the first brain regions impacted in dementia and Alzheimer's 37 disease, is important for transforming labile short-term memories (STM) to stable long-term 38 memories (LTM). Please provide an appropriate reference for this statement.

The authors seem to use rats in the early middle and old ages. However, the only mention under Subjects is that of rats aged 22-24 months old. Please change that to reflect the results section where rats aged 3, 12, and 24 are stated.

For the Statement: "Together, these data suggest that aging results in reductions in H2Bubi levels in the hippocampus that results in an incorrect response of this epigenetic mark following learning"- It is recommended to temper down the last bit of the sentence mentioning that reduction in h2bubi due to aging results in an incorrect response of this epigenetic mark following learning. Following fear learning there is no change in the H2bubi mark.

but not Manipulation (F(1,13) = 0.3068, P = 0.5891) and there was not a Time x Manipulation interaction- Please change the sentence to but not Manipulation and Time x Manipulation interaction.

Figure 1B please change the Y axis to OD% of Naïve aged and change the indicators as naïve aged and trained aged.

While the work is interesting and well written, there are a few points that should be considered prior to publication.

1. There is no information (e.g., ages, strain, and purchasing vendor) provided regarding the young control and middle-aged rats in the methods.

2. Fisher's LSD test does not typically correct for multiple comparisons and may not be the most appropriate post hoc test to follow up ANOVAs. This should be explained or switched to a more conservative post hoc test that corrects for multiple comparisons.

3. The methods provide no information regarding blinding or whether the data are appropriate for parametric tests, including whether this was assessed.

4. It would be helpful to either include young controls in some of measures, or provide information for how much of an increase is expected in H2Bubi after learning.

5. It is alluded that the lack of learning-induced increases in H2Bubi in the aged rat is because there is low Rnf20, but this is not measured. This appears to be the simplest and most testable interpretation, though other possibilities are considered in the Discussion

6. The data shown in supplementary figure 1 seems important, as it is the control to show that the Rnf20 manipulation increases H2Bubi levels. Yet, the stats are not clear. It looks the only increase in H2Bubi levels is relative to naïve (looks like there may be an outlier in the non-manipulated learning group). This should be explained and/or another measurement to show that H2Bubi levels are rescued by the Rnf20 manipulation.

7. It is unclear why only the naïve vs trained (Fig 3a) and naïve vs trained with Rnf20 were compared (3b). Wouldn't the better comparison be trained vs trained with Rnf20?

8. Fig 1: it would be easier for visualization if brackets or lines between the various comparisons were used (like in Fig 2) instead of "#" and "*" symbols. There is also a small typo in the figure legend title.

9. Fig 3 was particularly confusing in its representations. For instance, in Fig 3a,b, it would be helpful to provide information on the conditions being compared in the volcano plots. In all panels, it would also be helpful to use the same terms to describe the various conditions as in Fig 1 and 2.

10. While the hippocampus is largely affected by aging, it is not the only brain region affected. It would be helpful to provide information and/or speculate on the specificity or generality of these mechanisms for other brain regions and/or cognitive function.
